# Life Table Study of Fall Armyworm (*Spodoptera frugiperda*) (Lepidoptera: Noctuidae) on Three Host Plants under Laboratory Conditions

**DOI:** 10.3390/insects14040329

**Published:** 2023-03-28

**Authors:** Wen-Hua Chen, Brandon Itza, Lekhnath Kafle, Tsui-Ying Chang

**Affiliations:** 1 Department of Plant Medicine, National Pingtung University of Science and Technology, Pingtung 91201, Taiwan; 2 Department of Tropical Agriculture and International Cooperation, National Pingtung University of Science and Technology, Pingtung 91201, Taiwan

**Keywords:** fall armyworm, napier grass, natal grass, sunn hemp, alternative host plant

## Abstract

**Simple Summary:**

The fall armyworm, *Spodoptera frugiperda* (Lepidoptera: Noctuidae), is considered a lepidopteran pest that originated from the Americas. In 2019, the fall armyworm (FAW) was first reported in Taiwan. Alternative host plants also play an important role in the insect population ecology. Hence, the study aimed to investigate how different alternative host plants, two grasses (napier and natal grass) and one legume (sunn hemp) crop, affect the population growth of this insect pest. The findings revealed that alternative host plants significantly affect the developmental period and reproduction of this pest. Based on the age-stage, two-sex life table, sunn hemp resulted to be a more suitable host plant while natal grass a less suitable host plant for FAW. Overall, all alternative host plants could support the growth and development of this pest in the absence of the main host plant. As a result, monitoring the host plants for the pest in the off-season could be used as a technique for prediction.

**Abstract:**

After being discovered in Taiwan for the first time in June 2019, the polyphagous invasive fall armyworm (FAW), *Spodoptera frugiperda* (Lepidoptera: Noctuidae), has since spread throughout the entire nation. In Taiwan, this insect has a significant impact on the quality and output of wheat, corn, sorghum, and millet. It may further infest more crops in Taiwan due to its diverse range of hosts and alternate hosts. Maize and other staple crops have already been the subject of several study. The biology of FAW has not yet been studied in relation to the alternative hosts, particularly those commonly found in Taiwanese farmlands. Therefore, this study proposed to investigate the effects of napier grass (*Pennisetum purpureum*), natal grass (*Melinis repens*), and sunn hemp (*Crotalaria juncea*) on the development, reproduction, survivorship, and population growth of FAW under laboratory conditions. According to the results, the developmental duration was considerably the shortest when FAW was reared on sunn hemp while the longest on natal grass. Furthermore, female adults reared on napier grass had a longer adult pre-oviposition period, total pre-oviposition period, oviposition period, longevity, highest fecundity, and highest net reproductive rate (*R_o_*: 465.12). Among the tested three alternative host plants evaluated, sunn hemp had the highest intrinsic rate of increase (*r*: 0.1993), finite rate of increase (*λ*: 1.2206), and shortest mean generation time (*T*: 29.98). Therefore, this study suggests that all hosts plants can contribute to the development and outbreak of this pest in the absence of its primary host; however, sunn hemp was a relatively more suitable host plant for this insect. The possibilities for the FAW’s growth and development vary depending on the host plant. Thereby, all potential host plants in the area should be extensively examined while developing an IPM program against FAW.

## 1. Introduction

The fall armyworm (FAW), *Spodoptera frugiperda* (Lepidoptera: Noctuidae), is considered a major polyphagous lepidopteran pest that originated from the Americas [1]. In the American continent, FAW distribution was restricted till 2015. However, during 2016, an outbreak of FAW occurred in Central and Western Africa, and then across the entire continent of Southern Africa [2]. Due to its flight capacity, this transboundary pest has spread across the continent in less than two years [3]. By 2018, FAW was reported and confirmed in India [4] and by 2019, it spread across other Asian countries such as China, Thailand, Indonesia, the Philippines, Malaysia, Cambodia, Japan [5], and Taiwan [6]. In early 2020, FAW was confirmed in Australia, Timor Leste, Mauritania, and the United Arab Emirates [5]. At the end of 2020, countries with confirmed reports of invasion of FAW were Jordan, Papua New Guinea, and Syria [5]. During this present year, FAW has been confirmed in New Caledonia and its distribution from the Americas to over 70 countries is well-known [5].

Due to global concerns, FAO [5] mentioned that it’s important to consider the prevention measures against this pest. FAW possesses high migration ability, voracious larval feeding, high fecundity, and a wide range of hosts, all of which greatly contribute to causing economic damage to crop and pastures around the world [7]. According to reports from Africa, FAW might result in annual yield losses of approximately USD 10 billion in maize production [8]. In addition, Montezano et al. [9] and Rwomushana [10] reported that more than 350 plants from 76 different families are considered host plants of this pest. However, grasses such as sorghum and maize, which are regarded as their primary hosts, suffer the most damage [10]. Therefore, understanding and learning about the basic biological and ecological characteristics of the FAW are essential for developing successful management strategies [9]. 

In Taiwan, napiergrass is often used as cattle feed because of their preference and productivity. The Taishu No. 5 cultivar of Napiergrass predominates in Taiwan, where it covers approximately 3000 hectares throughout most of the counties. The primary cover crop or green manure crop, grown on approximately 200,000 hectares annually, is sunn hemp. In most of Taiwan’s counties, the local strain of sunn hemp is farmed. The Natal grass is an invasive weed and distributed all over Taiwan. Numerous studies on FAW’s primary hosts have been reported. In addition to the FAW, that has spread to all most all of Taiwan’s counties, natal grass, sunn hemp, and Napier grass are also widespread. All three plants mentioned previously also are related to known FAW host plants. The suitability of those plants for FAW to complete their life cycle must therefore be examined to understand how the alternative host plants affect the biological characteristics of the FAW [7]. This might facilitate the development of a better IPM strategy for combating the FAW. We therefore attempted to figure out whether three typical alternate hosts would be suitable for FAW to complete its life cycle. Thus, the objective of this study was to examine the effects of different alternative host plants, napier grass (*Pennisetum purpureum*), natal grass (*Melinis repens*), and sunn hemp (*Crotalaria juncea*) on the life history and demographic aspects of FAW under the laboratory conditions. 

## 2. Materials and Methods

### 2.1. Fall Armyworm Colony

Approximately 80 FAW larvae between the 3rd–5th instar stage were collected from maize plants grown at the National Pingtung University of Science and Technology (NPUST) practice farm, Pingtung, Taiwan. The collected larvae were transferred to the Tropical Agriculture Laboratory (rearing room) for the rearing of the stock colony under the laboratory climatic conditions. The larvae were individually placed in 6 cm Petri dishes and fed with an artificial diet [11] until pupation. Emerged adults were then paired inside acrylic cages (21H × 20W × 30L cm) with a nylon mesh cover for proper ventilation to the moths. Grass leaves and tissue paper strips were used as oviposition substrates for the collection of eggs. For the moths’ diet, a piece of damp cotton wool containing a 10% honey solution was placed inside the cages and replenished once a day. Protocols for stock colony establishment, host plant establishment, and life table study in this study were followed as proposed by Wang et al. [7] and Moraes et al. [12] with modifications.

### 2.2. Host Plants

Napier grass (Napiergrass Taishu No. 5) cuttings and natal grass (local strain) seedlings were collected from the NPUST Campus along with the purchase of sunn hemp (local strain) seeds. The host plants were successfully transferred and established in the Department of Plant Medicine greenhouse. The host plants were grown in plastic pots (18D × 14.7H cm) filled with a 1:1 soil/peat moss (Kekkilä substrate) mixture. All plants were kept and maintained throughout the completion of the experiments (napier grass: 25 March 2021 to 20 May 2021; natal grass: 25 June 2021 to 6 August 2021; sunn hemp: 13 November 2021 to 29 December 2021). At the beginning of the study, napier grass, natal grass, and sunn hemp plants were approximately 1 month old. Watering of the plants was carried out daily and fertilization with organic fertilizer (5-2-2, N:P:K) was performed at three-week intervals.

### 2.3. Life Table Study

Eight egg masses laid within 24 h were selected at random from the rearing room and transferred to the Plant Quarantine Laboratory at NPUST campus. A total of 12 eggs per egg mass were randomly selected and individually transferred to 150 mL plastic cups using a soft hairbrush. Moist cotton wool was placed inside the cups to help in maintaining a suitable relative humidity for the eggs (approximately 60–70% RH). The cups were then closed with plastic wrap and placed in a plant growth chamber with respective environmental conditions of 28 ± 1 °C, 70 ± 10% RH, and 12L:12D photoperiod. Whole experiments were conducted in the growth chambers, and the environmental conditions were kept the same throughout the entire experiment. The total numbers of eggs used were 95, 96, and 96 for napier grass (F2 generation, previously reared on artificial diet), natal grass (F4 generation, previously reared on artificial diet), and sunn hemp (F8 generation, previously reared on artificial diet), respectively. Every 24 h, the eggs were inspected, and the incubation duration and hatching rate were recorded. The larvae were then separately fed with excised leaves from each host plant and a piece of moist cotton wool was placed on the end of the leaf section to keep it from drying out. To prevent contamination during the experiment, the leaves were replaced with fresh leave sections daily until pupation. The individuals already in the pupa stage were collected every 24 h, sexed, and weighed on an electronic balance (Precisa 125 A). All larvae and pupae were maintained separately in the same plastic cup. The developmental duration at each stage and survivorship were recorded until the emergence of the moths. The emerging adult moths within the same host plants were mated and kept separately in 530 mL plastic cups. For the moths’ diet, the same procedure was used as described for the stock colony. Tissue paper strips were used as egg-laying substrates and lined on the interior walls of the cups. Whenever the adult male died, adult females were supplemented with adult males from the stock colony rearing room. Every day, the newly laid eggs were collected, and the daily egg production was recorded. The lifespan of the adults was also recorded until the last adult died.

### 2.4. Life Table Data Analysis

The life table raw data of FAW were analyzed according to the age-stage, two-sex life table [13,14,15] with the computer program TWOSEX-MSChart [16]. The age-stage specific survival rate (*S_xj_*); the age-specific survival rate (*l_x_*); the female age-specific fecundity (*m_x_*); the net reproductive rate (*R*_0_); the intrinsic rate of increase (*r*); the finite rate of increase (*λ*); and the mean generation time (*T*) were calculated according to Chi and Liu [15].

The age-stage specific survival rate (*S_xj_*) is defined as the probability that a newly laid egg will survive to age *x* and stage *j* (*x* is age in days and *j* is the stage). The age-specific survival rate (*l_x_*), i.e., the survival rate from age 0 to age *x*, was calculated as follows:

(1) $$l_{x}=\overset{m}{\underset{j=1}{\sum }}s_{xj}\;\;$$

The age-stage specific fecundity (*f_xj_*), i.e., the daily number of eggs laid by a female adult at age *x* and stage *j*, where *m* is the number of stages. The age-specific fecundity (*m_x_*), i.e., the average number of eggs produced by an individual at age *x*, was calculated as follows:

(2) $$m_{x}=\frac{\sum_{j=1}^{m}s_{xj}f_{xj}}{\sum_{j=1}^{m}s_{xj}}$$

The product of *l_x_* and *m_x_* is the age-specific net maternity (*l_x_m_x_*). The sum of *l_x_m_x_* over all ages gives the net reproductive rate (*R*_0_), i.e., the number of offspring produced by an individual during its lifetime, was calculated as follows:

(3) $$R_{0}=\overset{\infty }{\underset{x=0}{\sum }}l_{x}m_{x\;\;}$$

The intrinsic rate of increase (*r*) was calculated using the Lotka-Euler equation with age indexed from 0 [17] as follows:

(4) $$\overset{\infty }{\underset{x=0}{\sum }}e^{-r\left(x+1\right)}l_{x}m_{x}=1$$

The finite rate (*λ*) was calculated as follows:

(5) $$\lambda =e^{r}$$

The mean generation time (*T*) represents the period that a population is required to increase to *R*_0_-fold of its size as time approaches infinity and the population settles down to a stable age-stage distribution. The mean generation time was calculated as follows:

(6) $$T=\frac{\mathrm{In}\;R_{0}}{r}$$

### 2.5. Statistical Analysis

IBM SPSS statistics V22 was used for calculating the means and standard errors of the development duration, pupal weight, reproductive (oviposition and fecundity) parameters, and for comparative analysis among the host plants. The standard errors of APOP, TPOP, and population parameters were estimated by using the bootstrap technique [18,19] with 100,000 bootstraps. To determine the significance at *p* < 0.05 among the means, the Independent Sample *t*-test and One-way ANOVA followed by post hoc Tukey’s HSD methods were applied. Moreover, all the figures were prepared with SigmaPlot 14.0 software. 

## 3. Results

### 3.1. Developmental Duration, APOP, TPOP, Oviposition, Adult Longevity, and Fecundity of FAW

Regarding both sexes, the development of the same immature stage varies significantly among the tested host plants (1st instar: *F* = 15.750, *df* = 222, *p* < 0.001; 2nd instar: *F* = 3.207, *df* = 222, *p* = 0.008; 3rd instar: *F* = 6.085, *df* = 222, *p* < 0.001; 4th instar: *F* = 3.498, *df* = 222, *p* = 0.005; 5th instar: *F* = 4.261, *df* = 222, *p* < 0.001; 6th instar: *F* = 58.144, *df* = 222, *p* < 0.001; 7th instar: *F* = 1.922, *df* = 139, *p* = 0.095; 8th instar: *F* = 0.824, *df* = 9, *p* = 0.477; pupa: *F* = 80.351, *df* = 222, *p* < 0.001; egg to adult: *F* = 41.359, *df* = 222, *p* < 0.001). In addition, the developmental duration of male and female sexes on the same host plant differed significantly at the pupa and egg to the adult stage. Moreover, the developmental duration of both male and female sex was significantly shortest on sunn hemp at the egg to adult stage in comparison to the other hosts (Table 1).

When FAW was reared on the tested host plants, insect instars variation was observed. On napier grass, few of the individuals pupated in the 6th and 8th instar and the majority in the 7th instar. On natal grass, most of the individuals pupated in the 7th instar and a few in the 8th instar, respectively. As for sunn hemp, most of the individuals pupated in the 6th and few in the 7th instar.

Based on the results, the tested host plants significantly affected the reproduction capacity of FAW (APOP: *F* = 18.946, *df* = 97, *p* < 0.001; TPOP: *F* = 24.013, *df* = 97, *p* < 0.001; oviposition: *F* = 4.579, *df* = 97, *p* = 0.013). The APOP and TPOP were significantly longest when reared on napier grass than those reared on natal grass and sunn hemp, respectively. However, the significantly shortest TPOP was observed when reared on sunn hemp. In addition, the longest oviposition period was recorded on napier grass and was the shortest on natal grass. Based on the results, the tested host plants significantly affected the reproduction capacity of FAW (APOP: *F* = 18.946, *df* = 97, *p* < 0.001; TPOP: *F* = 24.013, *df* = 97, *p* < 0.001; oviposition: *F* = 4.579, *df* = 97, *p* = 0.013) (Table 1).

Furthermore, the results demonstrated that the longevity for both sexes significantly differed among the host plants (*F* = 12.46, *df* = 200, *p* < 0.001). Female and male longevity were significantly the longest when reared on napier grass and sunn hemp and the shortest when reared on natal grass. However, female and male longevity on the same host plant were not significantly different (Table 1).

FAW reared on different host plants exhibited a difference in its fecundity. The total numbers of eggs per female significantly varied from 1472.90 eggs on napier grass, 1185.59 eggs on sunn hemp, and 1021.19 eggs on natal grass (*F* = 6.405, *df* = 97, *p* = 0.002) (Table 1).

### 3.2. Pupal Weight of FAW

**Figure 1:**
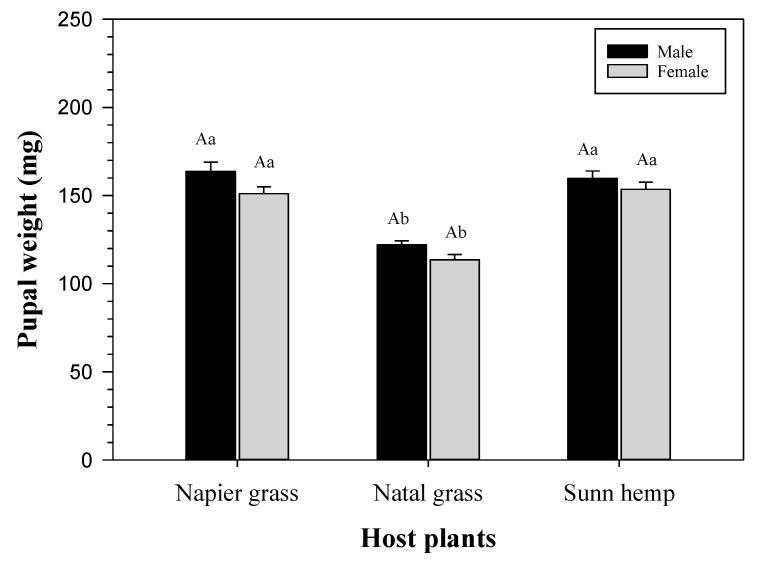
Pupal weight (mean ± SE) of FAW on napier grass (male, *n* = 31; female, *n* = 38), natal grass (male, *n* = 37; female, *n* = 43), and sunn hemp (male, *n* = 29; female, *n* = 45). Different uppercase letters (on the same host plant) or different lowercase letters (among the host plants) indicate significant differences (*p* < 0.05); One-way ANOVA: Tukey’s HSD.

Figure 1 shows that the pupal weight of both sexes differed significantly among the host plants. Both male and female pupae from napier grass and sunn hemp were noticeably heavier than those from natal grass (*F* = 30.074, *df* = 222, *p* < 0.001). However, the male and female pupal weights on the same host plant were not significantly different.

### 3.3. Life Table and Population Parameters of FAW

For the population parameters, the significantly highest intrinsic rate of increase (*r* = 0.1993), finite rate of increase (*λ* = 1.2206), lowest mean generation time (*T* = 29.98), and highest female sex ratio (0.46) were observed on sunn hemp. Furthermore, the net reproductive rate (*R*_0_ = 31.284) for FAW reared on napier grass was considered the highest (*r*: *F* = 71988.337, *df* = 299999, *p* < 0.001; *λ*: *F* = 72319.142, *df* = 299999, *p* < 0.001; *R*_0_: *F* = 43912.121, *df* = 299999, *p* < 0.001; *T*: *F* = 1410820.067, *df* = 299999, *p* < 0.001). Nevertheless, the lowest *r*, *λ*, *R*_0_ value, and lowest sex ratio was observed when FAW fed on natal grass (Table 2).

**Figure 2:**
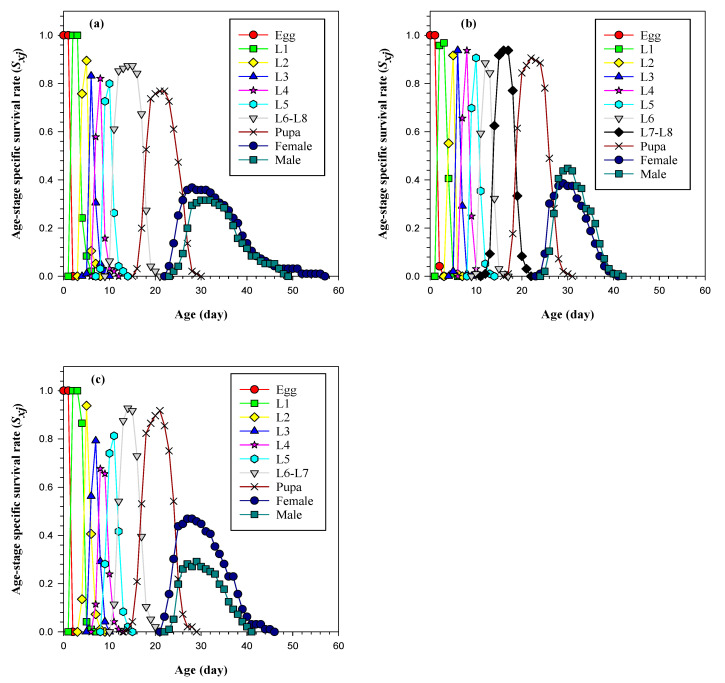
Age-stage specific survival rate of FAW on (**a**) napier grass, (**b**) natal grass, and (**c**) sunn hemp.

Based on the age-stage specific survival rate (*S_xj_*) curves of FAW, the highest larval survival rate was observed on natal grass as illustrated in Figure 2. Additionally, the lowest survival rate in the larval stage was recorded on sunn hemp followed by napier grass. The survival rate from the egg to the pupa stage was the highest on sunn hemp and natal grass, and the lowest on napier grass. Furthermore, the FAW population reared on natal grass had the highest adult survival rate, while the lowest was observed on napier grass.

**Figure 3:**
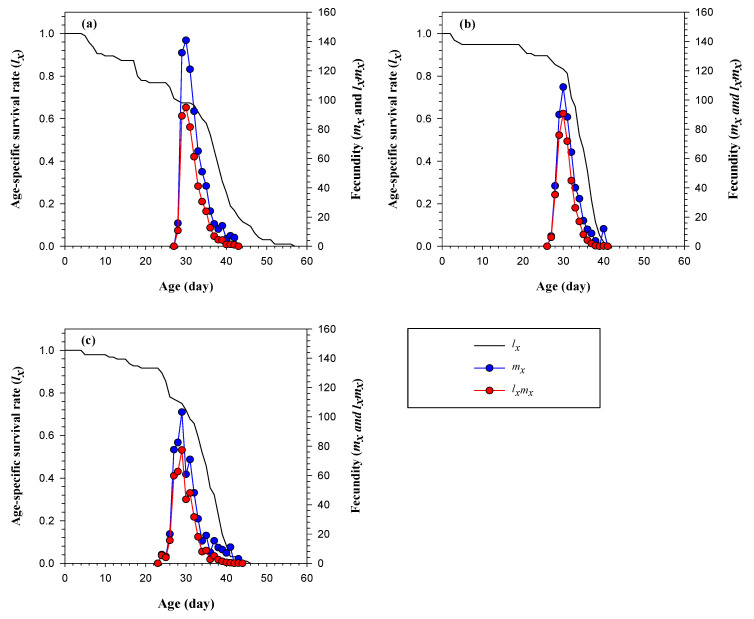
The age-specific survival rate (*l_x_*), the female age-specific fecundity (*m_x_*), and age-specific net maternity (*l_x_m_x_*) of FAW on (**a**) napier grass, (**b**) natal grass, and (**c**) sunn hemp.

The age-specific survival rate (*l_x_*) of FAW population gradually declined as the age (*x*) of the population increased. In the first 20 days, the *l_x_* curve diminished from 100% to 77%, 100% to 94%, and 100% to 91% on napier grass, natal grass, and sunn hemp, respectively. After day 20, the *l_x_* curve continued to steadily decline; however, a rapid decline on day 32 was recorded on natal grass and sunn hemp, indicating a high mortality in the adult stage (Figure 3). Figure 3 illustrates the female age-specific fecundity (*m_x_*) and age-specific net maternity value (*l_x_m_x_*) attained the reproductive high point at day 31, 31, and 30 on napier grass, natal grass, and sunn hemp, respectively. The highest *m_x_* value recorded on napier grass, natal grass, and sunn hemp was 140, 108, and 103, respectively. On napier grass and natal grass, the *m_x_* curve was demonstrated to have only one peak. However, on sunn hemp, more than one peak was observed in the *m_x_* curve, suggesting difference in the oviposition period of the individuals.

## 4. Discussion

Many study findings demonstrated that host plant species had a significant impact on insect life-history [20,21,22,23,24,25]. When an insect feeds on a certain host plant and exhibits a shorter developmental time and higher reproduction rates, the plant is considered to be more suitable [26]. Wang et al. [7] stated that FAW reared on its primary host, i.e., maize and wheat, develop faster and have heavier pupal weights. This study had similar results (data not included here) as claimed by Du Plessis et al. [27]; the development in the immature stage ranged from 25.93, 26.73, and 24.55 days on napier grass, natal grass, and sunn hemp, respectively, at 28 ℃. Furthermore, both male and female sex fed on sunn hemp had a significantly shorter egg to adult developmental duration than those reared on natal and napier grass. This can represent the higher suitability of the host plant for this insect. Moreover, the developmental duration of female in the pupa and egg to adult stage was significantly shorter than those of the male on the same host plant. Wang et al. [7] mentioned that this is considered as an attribute to the migratory characteristics of the insect since female adults need to emerge earlier than male adults to disperse, find a food source, and ensure a suitable oviposition site. 

In the present study, the number of insect instars before pupation varied from 6 to 8 instars when FAW was reared on the tested host plants. He et al. [23] and Pencoe and Martin [28] reported that the number of instars in FAW varied from 6 to 7 instars when reared on major oil crops (soybean and sunflower) and wild grasses (bahiagrass and yellow nutsedge). However, FAW necessarily needs 6 instars to complete the larval development [23]. In addition, it is believed that the number of insect instars could highly be affected by several factors such as temperature, inheritance, sex, food quality, and quantity [29]. Nevertheless, in the present study, food quality (host plants) might have had a greater influence on the number of insect instars since the environmental conditions were kept the same throughout the entire experiment. In addition, another factor might be that the larvae fed on their own head capsules. The collection of head capsules (molting) was necessary to determine the number of insect instars. Therefore, it is assumed that if the head capsule was consumed by the individual, then it can cause a variation in the number of insect instar.

Wang et al. [7] mentioned that plants species also influence the pupal weight of the FAW. They reported FAW reared on corn and wheat had a significantly heavier pupal weight compared to those reared on other cash crops such as tomato, soybean, and cotton. Similarly, the results of this study correlated with the results stated by Wang et al. [7] where the pupal weights of male and female did not differ significantly on the same host plant on certain plant species (wheat and soybean). Furthermore, the present findings are in accordance with the findings of He et al. [23,25] and demonstrated that the pupal weight of males was noticeably higher than the pupal weight of females on the tested host plants. According to the claims of He et al. [23], the characteristics in the pupal stage are attributed to factors such as the fitness and nutritional status of the larvae. Based upon observations, FAW larvae reared on napier grass were bigger and reflected in having heavier pupal weights compared to those reared on natal grass.

Pencoe and Martin [28] reported that different food plants showed to have effects on the reproductive capacity of the insect. According to Wu et al. [30], insects feeding on host plants with low nutritional value may lead to smaller individuals, declined egg development, and a reduced reproduction capacity. With these findings, it is considered that napier grass and sunn hemp had a higher nutritional value compared to natal grass. Therefore, the results of this study demonstrated and supported previous findings and showed a positive relationship between the pupal weight and the reproduction of the insect. FAW fed on napier grass showed a significantly longest APOP (4.63) and TPOP (29.83), while the shortest TPOP (27.63) was recorded on sunn hemp. Xie et al. [21] claimed that there was no significant difference on the APOP when FAW was reared on maize and kidney bean; however, the significantly shortest TPOP was recorded on maize while the longest was observed on kidney beans. Wu et al. [30] showed that FAW reared on maize demonstrated the shortest APOP and TPOP compared to other host plants (tomato and pepper). On both mentioned studies, a shorter APOP and TPOP was recorded on the most suitable host plant. Moreover, the longest oviposition (8.20), adult longevity (female: 14.40; male: 12.54), and highest fecundity (1472.90) were observed on napier grass followed by sunn hemp. Our research revealed that not all grasses are favorable for FAW growth and development and reproduction. Regarding the oviposition period and fecundity, a recent study showed that legumes are considered a suitable source for FAW. Gebretsadik et al. [31] reported that FAW fed on maize and faba beans had longer oviposition periods and higher fecundities in comparison to other hosts such as wheat and barley. In accordance with Barcelos et al. [32], heavier pupa might produce bigger and stronger adults, which might increase the insect’s reproduction capacity. He et al. [23] argued that adult fecundity is mainly relied on two factors, the accumulated nutrition in the immature (larval) stage and the adult stage. However, in the present study, all adults were fed with 10% honey solution, it is believed that the host plant mainly contributed to the change in the reproduction capacity of this pest.

The life table parameters are not only affected by host species but often vary with chemical pesticides and environmental conditions [20,33]. As demonstrated by Wang et al. [7], FAW reared on maize had a higher population increase capacity (higher *r*, *λ*, *R*_0_, and lower *T*) compared to tomato, cotton, and soybean. Wu et al. [30] reported similar results on maize, except tomato resulted with the highest net reproductive rate. The findings of this study had similar results on sunn hemp (higher *r*, *λ*, and lower *T*) as reported on maize by previous authors. Although FAW reared on sunn hemp and napier grass showed the lowest larval survival rate, its high female fecundity made a remarkable contribution in obtaining a high *R*_0_ (napier grass: 465.12; sunn hemp: 395.19). The lowest *R*_0_ (382.94) value was recorded on natal grass due to a lower fecundity, indicating that this host plant negatively affected the reproduction of FAW. Consequently, other host plants such as rice and potato have shown to negatively affect the reproduction of FAW by obtaining a lower *R*_0_ value [34]. According to Acharya et al. [34], FAW resulted in having a shorter preadult developmental time, higher preadult survival rate, shorter APOP and TPOP, longer adult longevity, higher mean fecundity, and greater pupa weight when reared on maize. As a result of the combined effects of the mentioned parameters, FAW reared on maize had a higher population increase capacity (higher *r*, *λ*, *R*_0_ and shorter *T*) than those reared on the other host plants (rice and potato). Therefore, an extended preadult developmental time, lower pupal weight, longer TPOP, shorter adult longevity, and lower fecundity observed on natal grass resulted in having lower *r*, *λ*, *R*_0_ values. In addition, Guo et al. [35] emphasized the importance of the female ratio since females contribute principally to gene flow and dispersal of a population. Although the female fecundity recorded on sunn hemp was not the highest among the tested host plants, FAW reared on sunn hemp was demonstrated to obtain the highest female ratio (0.46) among the hosts.

The different host plants were examined during different time periods. Apart for a few external components, all the host plants’ growing conditions in the greenhouse were maintained the same across all the hosts. Furthermore, the ambient parameters were maintained constant during the entire experiment, and all experiments were carried out in growth chambers, though we must acknowledge that there is a chance for this confound.

The agricultural practices such as push–pull or maize–legume intercropping have been shown to drastically minimize FAW infestations. The precise mechanisms governing FAW management, however, have not yet been explained in detail. Hence, using three host plants that are often used in push–pull crop intercropping, we assessed the growth and development of the FAW, the adult reproductive rate, the adult longevity, and the adult survival rate. The sun hemp could be used as a trap crop in a push–pull system because it is one of the highly preferred hosts.

## 5. Conclusions

The alternative host plants affect the life history and life table parameters of FAW. FAW reared on napier grass demonstrated a shorter developmental duration, higher reproduction capacity, lower preadult survival rate, higher female ratio, and higher population growth capacity (only *R*_0_). FAW reared on natal grass demonstrated a longer developmental duration, lower reproduction capacity, higher preadult survival rate, lower female ratio, and lower population growth parameters (except *T*). FAW reared on sunn hemp demonstrated the shortest developmental duration, high reproduction capacity, lowest preadult survival rate, highest female ratio, and highest population growth parameters (*r*, *λ*, and lowest *T*). Considering the results obtained in this study, sunn hemp was a more suitable host followed by napier grass, while natal grass a less suitable host plant for fall armyworm. Nevertheless, this study observed that, in the absence of its primary host, the tested alternative host plants could support the establishment and development of the FAW population and contribute to the spreading of the pest. Out of the three host plants and regarding their suitability to FAW, natal grass being the least favorable host plant could be most likely used to suppress the pest population growth. Monitoring the population of this pest on these alternative host plants during the off-season could possibly be used as a prediction tool for risk assessments to nearby farms. Nonetheless, further study of this pest under field condition is needed for a better understanding of its population ecology. 

## Figures and Tables

**Table 1:** Developmental duration, APOP, TPOP, oviposition, adult longevity, and fecundity (mean ± SE) of FAW on different host plants.

Stage	Sex (*n*)	Napier Grass *	Sex (*n*)	Natal Grass	Sex (*n*)	Sunn Hemp
Egg	♀ (38)	2.00 ± 0.00 Aa	♀ (37)	2.00 ± 0.00 Aa	♀ (45)	2.00 ± 0.00 Aa
	♂ (31)	2.00 ± 0.00 Aa	♂ (43)	2.00 ± 0.00 Aa	♂ (29)	2.00 ± 0.00 Aa
1st instar	♀ (38)	2.24 ± 0.08 Ab	♀ (37)	2.38 ± 0.08 Ab	♀ (45)	2.84 ± 0.07 Aa
	♂ (31)	2.16 ± 0.06 Ab	♂ (43)	2.47 ± 0.08 Ab	♂ (29)	2.93 ± 0.04 Aa
2nd instar	♀ (38)	1.79 ± 0.06 Aa	♀ (37)	1.59 ± 0.08 Aa	♀ (45)	1.53 ± 0.07 Aa
	♂ (31)	1.90 ± 0.07 Aa	♂ (43)	1.53 ± 0.07 Ab	♂ (29)	1.66 ± 0.12 Aab
3rd instar	♀ (38)	1.39 ± 0.08 Ab	♀ (37)	1.32 ± 0.07 Ab	♀ (45)	1.76 ± 0.06 Aa
	♂ (31)	1.35 ± 0.08 Aab	♂ (43)	1.35 ± 0.07 Ab	♂ (29)	1.69 ± 0.08 Aa
4th instar	♀ (38)	1.74 ± 0.07 Aa	♀ (37)	1.92 ± 0.05 Aa	♀ (45)	1.69 ± 0.07 Aa
	♂ (31)	1.71 ± 0.08 Aa	♂ (43)	1.98 ± 0.04 Aa	♂ (29)	1.69 ± 0.08 Aa
5th instar	♀ (38)	2.08 ± 0.08 Aa	♀ (37)	2.11 ± 0.06 Aa	♀ (45)	2.36 ± 0.07 Aa
	♂ (31)	2.13 ± 0.07 Ab	♂ (43)	2.16 ± 0.05 Ab	♂ (29)	2.48 ± 0.09 Aa
6th instar	♀ (38)	2.76 ± 0.19 Ab	♀ (37)	2.81 ± 0.06 Ab	♀ (45)	4.64 ± 0.10 Aa
	♂ (31)	2.68 ± 0.21 Ab	♂ (43)	2.81 ± 0.06 Ab	♂ (29)	4.83 ± 0.11 Aa
7th instar **	♀ (31)	4.74 ± 0.07 Aa	♀ (37)	4.35 ± 0.16 Aa	♀ (2)	5.00 ± 0.00
	♂ (26)	4.73 ± 0.16 Aa	♂ (43)	4.65 ± 0.10 Aa	♂ (1)	6.00 ± 0.00
8th instar ***	♀ (0)	-	♀ (5)	4.40 ± 0.24 A	♀ (0)	-
	♂ (2)	4.50 ± 0.50 a	♂ (3)	4.00 ± 0.00 Aa	♂ (0)	-
Pupa	♀ (38)	7.26 ± 0.08 Ba	♀ (37)	7.03 ± 0.06 Bab	♀ (45)	6.96 ± 0.07 Bb
	♂ (31)	8.71 ± 0.09 Aa	♂ (43)	8.02 ± 0.06 Ab	♂ (29)	7.93 ± 0.06 Ab
Egg to adult	♀ (38)	25.13 ± 0.21 Bb	♀ (37)	26.11 ± 0.17 Ba	♀ (45)	24.00 ± 0.18 Bc
	♂ (31)	26.90 ± 0.21 Aa	♂ (43)	27.26 ± 0.16 Aa	♂ (29)	25.41 ± 0.24 Ab
APOP	♀ (30)	4.63 ± 0.23 a	♀ (36)	3.03 ± 0.15 c	♀ (32)	3.84 ± 0.16 b
TPOP	♀ (30)	29.83 ± 0.20 a	♀ (36)	29.11 ± 0.19 a	♀ (32)	27.63 ± 0.27 b
Oviposition	♀ (30)	8.20 ± 0.46 a	♀ (36)	6.17 ± 0.40 b	♀ (32)	6.78 ± 0.57 ab
Adult longevity	♀ (30)	14.40 ± 0.88 Aa	♀ (36)	9.41 ± 0.39 Ab	♀ (32)	11.78 ± 0.56 Aab
	♂ (31)	12.55 ± 0.82 Aa	♂ (43)	8.67 ± 0.42 Ab	♂ (29)	10.31 ± 0.57 Aab
Fecundity (eggs/♀)	♀ (30)	1472.90 ± 108.38 a	♀ (36)	1021.19 ± 69.49 b	♀ (32)	1185.59 ± 93.76 ab

* Means in the same row and column within the same developmental stage followed by different lowercase letters (among the host plants) or different uppercase letters (on the same host plant) indicate significant differences (*p* < 0.05); One-way ANOVA: Tukey’s HSD. ** No comparative analysis was conducted for individuals reared on sunn hemp due to the low sample size number. *** Individuals (female sex) reared on napier grass did not go through the 8th instar. Therefore, no statistical analysis was conducted at the 8th instar stage on napier grass. Comparative analysis was still conducted for individuals (male sex) reared on napier and natal grass using the Independent Sample *t*-test.

**Table 2:** Population parameters (mean ± SE) and female ratio of FAW on different host plants.

Host Plants	Population Parameters				
	*r* (Day^−1^) *	*λ* (Day^−1^)	*R*_0_	*T* (Day)	Female Ratio
Napier grass	0.1918 ± 0.0058 b	1.2114 ± 0.0070 b	465.12 ± 77.88 a	32.02 ± 0.24 a	0.40
Natal grass	0.1901 ± 0.0049 c	1.2094 ± 0.0060 c	382.94 ± 56.67 c	31.28 ± 0.18 b	0.38
Sunn hemp	0.1993 ± 0.0062 a	1.2206 ± 0.0076 a	395.19 ± 64.64 b	29.98 ± 0.36 c	0.46

* Means in the same column followed by different lowercase letters are significantly different (*p* < 0.05); One-way ANOVA, Tukey’s HSD.

## Data Availability

The data presented in this study are available on request from the corresponding author. The data are not publicly available due to the privacy issues of funding agencies.
